# Electrospun Fenoprofen/Polycaprolactone @ Tranexamic Acid/Hydroxyapatite Nanofibers as Orthopedic Hemostasis Dressings

**DOI:** 10.3390/nano14070646

**Published:** 2024-04-08

**Authors:** Chang Huang, Menglong Wang, Siyou Yu, Deng-Guang Yu, Sim Wan Annie Bligh

**Affiliations:** 1 School of Materials and Chemistry, University of Shanghai for Science and Technology, Shanghai 200093, China; 213353177@st.usst.edu.cn (C.H.); 191370148@st.usst.edu.cn (M.W.); 2135051725@st.usst.edu.cn (S.Y.); 2 School of Health Sciences, Saint Francis University, Hong Kong 999077, China

**Keywords:** modified coaxial electrospinning, core–shell nano hybrids, hemostasis, multiple functions, orthopedic dressings

## Abstract

Dressings with multiple functional performances (such as hemostasis, promoting regeneration, analgesia, and anti-inflammatory effects) are highly desired in orthopedic surgery. Herein, several new kinds of medicated nanofibers loaded with several active ingredients for providing multiple functions were prepared using the modified coaxial electrospinning processes. With an electrospinnable solution composed of polycaprolactone and fenoprofen as the core working fluid, several different types of unspinnable fluids (including pure solvent, nanosuspension containing tranexamic acid and hydroxyapatite, and dilute polymeric solution comprising tranexamic acid, hydroxyapatite, and polyvinylpyrrolidone) were explored to implement the modified coaxial processes for creating the multifunctional nanofibers. Their morphologies and inner structures were assessed through scanning and transmission electron microscopes, which all showed a linear format without the discerned beads or spindles and a diameter smaller than 1.0 μm, and some of them had incomplete core–shell nanostructures, represented by the symbol @. Additionally, strange details about the sheaths’ topographies were observed, which included cracks, adhesions, and embedded nanoparticles. XRD and FTIR verified that the drugs tranexamic acid and fenoprofen presented in the nanofibers in an amorphous state, which resulted from the fine compatibility among the involved components. All the prepared samples were demonstrated to have a fine hydrophilic property and exhibited a lower water contact angle smaller than 40° in 300 ms. In vitro dissolution tests indicated that fenoprofen was released in a sustained manner over 6 h through a typical Fickian diffusion mechanism. Hemostatic tests verified that the intentional distribution of tranexamic acid on the shell sections was able to endow a rapid hemostatic effect within 60 s.

## 1. Introduction

Bone injury is caused by many reasons, including traumatic injury (fracture and hairline fracture), deformity of the back and neck, arthrophlogosis, and muscle tendinitis [1]. When an injury happens to the osseous tissue, such as a fracture or light defects, osseous tissue will proceed with its self-healing and remodeling process to recover its function through intramembrane and endochondral ossification occurs at the site of injury. However, some severe bone defects comprising traumatic injuries, degenerative diseases, tumor resections, infections, and birth defects may need clinical interventions to promote a complete recovery. During the clinical interventions of orthopedic surgery, the risk of bleeding is usually accompanied. Thus, controlling bleeding in the orthopedic surgery process and the elimination of other risks (such as pain and infection) is always a big concern [2]. In clinics, there are some traditional methods of controlling bleeding, such as mechanical (compression and suture), heat (laser), and medicated materials. One of the popular medicated materials is bone wax, which is commonly used for bleeding control in orthopedic surgery because of its satisfactory bone viscosity and ductility [3]. But, it has significant limitations, such as hydrophobic properties, and is prone to cause complications. Tan et al. [4] reported a class of composite materials to replace bone wax that could attach to the bleeding bone as tamponade with low cytotoxicity, biodegradability, and coagulation hemostatic sealing potential. However, it is difficult to control bone surface bleeding and intramedullary canal bleeding. An ideal orthopedic dressing should be endowed with an effective hemostatic effect to reduce blood transfusion rate, as well as prevent ectopic ossification, enhance postoperative anticoagulation, and relieve pain for a final joint effect of realizing quick bone recovery [5].

In this nano era, nanotechnologies and the related nanomaterials have demonstrated their powers in almost all the applied scientific fields [6,7], including numerous biomedical treatments [8,9,10,11,12,13,14]. Those nano-related elements are bringing out revolutionary changes in the field of orthopedics for orthopedic surgery. Their reasonable applications have been demonstrated to be able to promote hemostatic control of orthopedic surgery, improve surgical outcomes, and have the effect of promoting bone healing and reducing the appearance of complications after orthopedic surgery [15]. Among all types of nanotechnologies and nanomaterials, electrospinning and electrospun nanofibers are very special due to the single-step top–down fabrication process and the unique properties of the resultant fibrous mats [16,17,18,19]. These properties include high specific surface area and porosity [20,21], 3D web structure, and facile to be modified [22,23,24,25]. Today, electrospinning is differentiated into many subbranches [26,27,28,29,30] and is expanding its real applications in many regions [31,32,33]. The monoaxial single-fluid electrospinning is the simplest and most basic one [34,35,36,37,38]. Typically, a single-fluid electrospinning can be successfully conducted with a spinnable solution with enough polymeric chain entanglements. However, for the multifluid process, unspinnable solutions can also be treated as long as one of them is electrospinnable [39,40,41]. The interfacial tensions between the spinnable and unspinnable solutions are able to play their roles in guiding those solutions to form the designed multichamber nanostructures [42,43,44,45]. These multiple-chamber nanostructures include bichamber core–shell and side-by-side (or Janus), trichamber core–shell and trisection side-by-side, and trichamber combinations of core–shell and Janus. No matter how complex the nanostructures are, they can be directly created in a single step and straightforward manner, which is impossible for many bottom–up chemical synthesis methods.

The modified coaxial electrospinning for preparing core–shell nanofibers is one of the most popular one among all multifluid electrospinning processes due to the usage of an unspinnable shell fluid and the popularity of core–shell nanostructures [46]. For a reasonable selection of the core electrospinnable polymeric matrix to develop core–shell biomedical products, the compatibility, solubility, biodegradable, and mechanical properties should be taken into consideration. Polycaprolactone (PCL) is a kind of biodegradable material [47,48,49] and has been widely used as tissue engineering scaffolds [50,51,52]. Elangomannan et al. [53] reported a carbon nanofiber/PCL/mineralized hydroxyapatite fibrous scaffold coated on the titanium for corrosion resistance purposes. This coating mimicked natural bone with good mechanical properties and biocompatibility and has potential in orthopedic applications. Another advantage of using electrospinning is that it is an easy method of fabricating a drug delivery system, by which the drug molecules can be facilely encapsulated into the nanofibers, and their release behaviors are easily controlled [54,55]. As early as 2002, the first drug delivery system based on electrospun nanofibers was reported [56].

Based on the above-mentioned knowledge about the requests of an ideal orthopedic dressing for multiple functional performances and the capability of electrospinning in preparing composite medicated nanomaterials, we hypothesized that multiple active ingredients could be encapsulated into the electrospun core–shell PCL structure, and by utilizing a modified coaxial electrospinning technique, the distribution of these ingredients can be controlled, potentially leading to a synergistic effect on both bleeding control and bone wound healing.

## 2. Materials and Methods

### 2.1. Materials

Polyvinylpyrrolidone K30 (PVP K30, *M*_w_ ~ 40,000) was purchased from Sigma Aldrich (Shanghai, China). Polycaprolactone (PCL, *M*_w_ ~ 80,000), tranexamic acid (TA, 98%), hydroxyapatite (HAP, ≥97%), 2-(3-Phenoxyphenyl)propionic acid calcium salt dihydrate (fenoprofen, ≥98%), and FeCl_3_·6H_2_O (99%) were obtained from Shanghai Macklin Biochemical Co., Ltd. (Shanghai, China). Solvents, including trifluoroethanol (TFE), dichloromethane (DCM), and ethyl alcohol (EtOH), were provided by Sinopharm Chemical Regent Co., Ltd. (Shanghai, China), and they are all analytical reagents. Water was double-distilled just before usage.

### 2.2. Conducting Modified Coaxial Electrospinning

Several working solutions were prepared as follows: In total, 1.0 g PCL and 0.4 g fenoprofen were codissolved into a mixed solvent comprising DCM and EtOH with a 9:1 ratio in volume and named solution A. A total of 0.1 g tranexamic acid and 0.1 g hydroxyapatite were dispersed into 10 mL TFE as the solution B. Both monoaxial and modified coaxial electrospinning processes were conducted under room temperature (20 ± 3 °C) and relative humidity of (60 ± 5)%. The applied voltage and collected distance were fixed at 12.0 kV and 20 cm. The flow rate of each layer and morphologies of all the products are included in Table 1.

### 2.3. Modified Coaxial Electrospinning Processes

A homemade coaxial electrospinning apparatus was used for all the preparations. The apparatus was mainly composed of two pumps (KDS 100, KD scientific, Vernon Hills, IL, USA), a self-made concentric spinneret, a high voltage supply (ZGF2000/6mA, Wuhan Hua-Tian High Power Co., Ltd., Wuhan, China) and a homemade receiving device. The general procedures for implementation are listed as follows: (1) The prepared different working fluids were loaded into 20 mL syringes, respectively, and correctly fixed on the two injection pumps. (2) The syringe and the coaxial spinneret were connected together, and the alligator clamp was fixed to convert the voltage from the high voltage supply. (3) Under the push of the injection pumps, the working fluids were driven out to the tip of the spinneret to form a droplet. (4) After the working fluids were pushed out continuously, the high voltage supply was switched on, and the applied voltage was slowly increased. (5) When the applied voltage was high enough to overcome the surface tension of the droplet, a Taylor cone was formed at the tip of the spinneret, following which a straight fluid jet was emitted out and began the continuous and successive bending and whipping loops. (6) The solidified nanofibers are deposited on the collector in a random manner. The collected nanofibers were stored in a drying oven until constant weight.

### 2.4. Characterization

#### 2.4.1. Morphologies and Inner Chambers

Morphologies of the prepared nanofibers were investigated through scanning electron microscopy (SEM, Quanta FEG450, FEI Corporation, Hillsboro, OR, USA) after samples had been sputtered gold with 5 nm thickness by high vacuum turbo evaporator (Q150TES, Quorum, UK). The diameters of nanofibers were counted through ImageJ software (National Institutes of Health, Bethesda, MD, USA) by randomly picking 100 places in SEM images, and the diameter distribution was automatically provided by this software. The inner structures of F3 and F4 were evaluated using transmission electron microscopy (TEM, JEM 2200F, JEOL, Tokyo, Japan).

#### 2.4.2. Physical State and Compatibility

Physical states of raw materials and obtained nanofibers were detected by X-ray diffraction (XRD, Bruker, Bremen, Germany). The data were collected from 10° to 70° with 3° per minute. Fourier transform infrared spectroscopy (FTIR, Spectrum 100, Perkin-Elmer, Waltham, MA, USA) tests of raw materials and nanofibers were conducted to explore the chemical compatibility within nanofibers. Absorption intensity among 500 cm^−1^~4000 cm^−1^ was gathered with the resolution of 4 cm^−1^. Each spectrum was obtained as an average of 8 scans.

#### 2.4.3. Hydrophilic Properties

The water contact angle (WCA) tests were conducted using an interfacial tension measuring apparatus (JC2000C1, Shanghai Zhongchen Digital Technology Apparatus Co., Ltd., Shanghai, China). Each group of samples to be tested was cut into a suitable size rectangle (60 × 10 mm^2^) and pasted on the slide, and the slide was placed on the work table. Deionized water was selected as the probe liquid, and the contact angle of water was measured by the droplet method. A total of 5 μL of deionized water was dropped on the under-testing membrane. The wetting processes were recorded by the inbuilt high-speed camera. For each sample, the process was conducted six times, and the data were recorded as mean ± S.D. WCAs were measured by ImageJ using a plugin named drop analysis.

#### 2.4.4. In Vitro Drug Release Profiles

Drug release profiles of different medicated fibers were explored by in vitro experiments. A 0.1 g sample membrane was put into the beaker with 450 mL of deionized water. Meanwhile, the beaker was placed in the shaking bath with 37 °C and 50 rpm shaking speed. To measure the level of fenoprofen, a 4 mL sample was withdrawn at a predetermined time from the beaker, and the same volume of deionized water was added. The concentration of fenoprofen was detected by UV-vis spectrophotometry according to the preacquired standard curve. The cumulative release rate can be calculated using the following equation:

(1) $$P\left(\%\right)=\frac{V_{0}c_{n}+\sum_{i=1}^{n-1}c_{i}V}{Q}\times 100$$

where *c_n_* and *c_i_* are calculated concentrations at *n* and *i* times; *V*_0_ is the volume of water in conducting in vitro experiments; *V* is the volume withdrawn each time; *Q* represents the theoretical drug mass in membrane; and *P* is the release rate of drug.

#### 2.4.5. In Vitro Hemostatic Tests

In vitro hemostatic time tests were conducted by adding fresh blood tokens from rats into different groups of prepared fibers. Before the experiment, a certain amount of anticoagulant blood should be prepared for later use. Among them, the preparation method of anticoagulation was as follows. A 1 mL sterile syringe for blood collection and a 10 mL centrifuge tube for backup anticoagulation were injected with sodium citrate anticoagulant before blood collection. In total, 1 mL of anticoagulant was inhaled into a sterile syringe, 10 mL of anticoagulant was added into the centrifuge tube, and then the anticoagulant was poured out and repeated three times. After anesthesia, the blood was taken from the eyelid venous plexus with a sterile syringe and injected into a centrifugal tube to shake well so that the blood was fully in contact with the anticoagulant and mixed to prepare anticoagulant blood for use. An amount of 5.0 mg of each fiber sample was weighed and put into a 1.5 mL type centrifuge tube. Then, 0.5 mL of anticoagulant blood was dropped into a centrifuge tube quickly. After 3 s vortex, the centrifuge tube was put into a 37 °C water bath. The centrifuge tube was inverted every 15 s, and coagulation conditions were recorded by photos.

## 3. Results and Discussion

### 3.1. Selections of Raw Components for Fabricating the Orthopedic Dressings

Reasonable selections and combinations of the starting components are very important for developing functional nanoproducts [10,11,12,13,14]. As a hemostatic medicine recommended by the World Health Organization (WHO), tranexamic acid (TA) can be used as an antifibrinolytic agent [57,58]. This medicine can promote hemostasis by directly preventing plasminogen activation [59,60,61]. At the same time, it has a strong growth inhibitory effect on some Gram-positive bacteria and Gram-negative bacterial strains [62]. Nqoro et al. [63] synthesized a local hemostatic gel encapsulated with TA and metal nanoparticles, which exhibited excellent cell viability, antibacterial properties, and hemostatic ability and could be used as a scaffold that could control bleeding and microbial infection of wounds. Electrospun TA-loaded composite nanofibers from single-fluid electrospinning for hemostatic applications have been reported previously [64,65,66,67]. Varshosaz et al. [67] reported a kind of nanofibrous membranes containing polyglycerol sebacate and polyhydroxyethyl methacrylate and a small amount of TA for a fast hemostatic effect. However, it is hard to furnish these nanofibers with other functional performances due to a monoaxial nanostructure and a sole active ingredient.

Hydroxyapatite (HAP) is a commonly used biocompatible and biodegradable material [68,69]. HAP has major components similar to biological apatite [70], which is a natural mineral part of bone tissue [71]. This material has a great synostosis ability, which can not only promote cell adhesion, proliferation, and extracellular matrix secretion but also has the ability to form bonds with bone in the body and to help repair bone defects [72,73,74]. Thus, HAP has already been widely used as a biomaterial and applied to promote the recovery of damaged and diseased bone tissue [75]. However, HAP is not easily processed into implantation materials [76]. To take advantage of these materials, many strategies have been proposed. For example, Sheikh et al. [77] prepared HAP particles embedded in nanofibers. Wu et al. [78] grew HAP on the carbonized poly(acrylonitrile) nanofibers, which were prepared through a monoaxial electrospinning process. HAP is, therefore, widely used in bone injury repair. Yang et al. [79] prepared a new hyaluronic-alginate three-dimensional scaffold containing HAP and titanium nanoparticles by freeze-drying, giving a hint of a new idea for repairing bone injury.

There is also a problem of bacterial infection that cannot be ignored in orthopedic hemostatic applications. *Staphylococcus aureus* (*S. aureus*) is a pathogenic microorganism that can cause serious bone infections. The presence of *S. aureus* could promote the death of osteoblasts and differentiation of osteoclasts in bone infection, finally leading to the adverse consequences of bone homeostasis imbalance [80]. Fenoprofen, a nonsteroidal anti-inflammatory drug [81], is usually used to relieve the pain from osteoarthritis and rheumatoid arthritis. Moreover, fenoprofen has excellent cell biocompatibility when the concentration is less than 100 μM and does not cause cell killing. In addition, Jiang et al. [82] also found in related studies that fenoprofen weakened *S. aureus* and inhibited osteolysis caused by *S. aureus*. When used in vivo for treatment, fenoprofen promoted the recovery of walking ability of osteomyelitis mice by relieving pain and eliminating bacterial infection.

**Figure 1:**
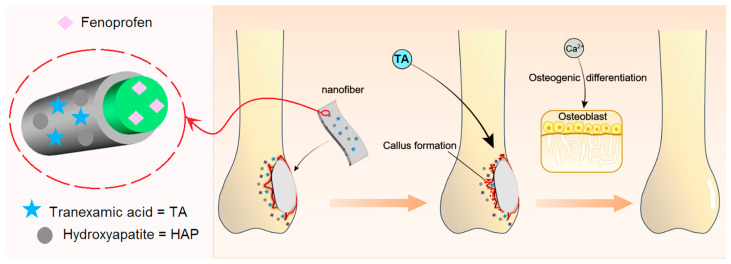
The designed electrospun orthopedic dressing composed of fenoprofen/PCL @ TA/HAP core–shell nanofibers for a joint repair of bone injury.

Thus, based on the electrospun core–shell nanostructure, we selected TA, HAP, and fenoprofen as the active ingredients to be coencapsulated within the designed orthopedic dressing. To achieve a combined impact on both bleeding control and healing of a bone wound, we have strategically planned the deliberate distributions of these ingredients within the electrospun fenoprofen/PCL @ TA/HAP nanofibers (Figure 1). The core sections of the electrospun nanofibers consist of PCL and fenoprofen, which enable a sustained release of analgesia and anti-infection properties over an extended period. In contrast, the shell section containing TA is designed for immediate release, facilitating a rapid hemostatic action. Additionally, the insoluble HAP nanoparticles adhering to the surface of PCL nanofibers gradually release their molecules, promoting the proliferation of osteoblasts.

### 3.2. Reasonable Implementations of the Modified Coaxial Electrospinning

Traditionally, coaxial electrospinning is regarded as a process in that the shell fluid must be electrospinnable, whereas the core fluid can be or cannot be spinnable for creating the core–shell nanostructure [20]. Yu et al. broke this concept to develop modified coaxial electrospinning, which was characterized by the exploitation of unspinnable fluids as the shell-working fluids for creating core–shell nanofibers [45,47]. On the one hand, the extension of shell fluids from the electrospinnable solutions to the unspinnable fluids would greatly expand the capability of electrospinning in creating novel core–shell nanofibers because the numbers of unspinnable fluids are far more than those electrospinnable ones. On the other hand, the influence of different kinds of unspinnable working fluids on the working processes and the resultant nanofibers receive no attention in the literature. Based on this situation here, various types of unspinnable shell fluids were prepared to implement the modified coaxial electrospinning processes.

**Figure 2:**
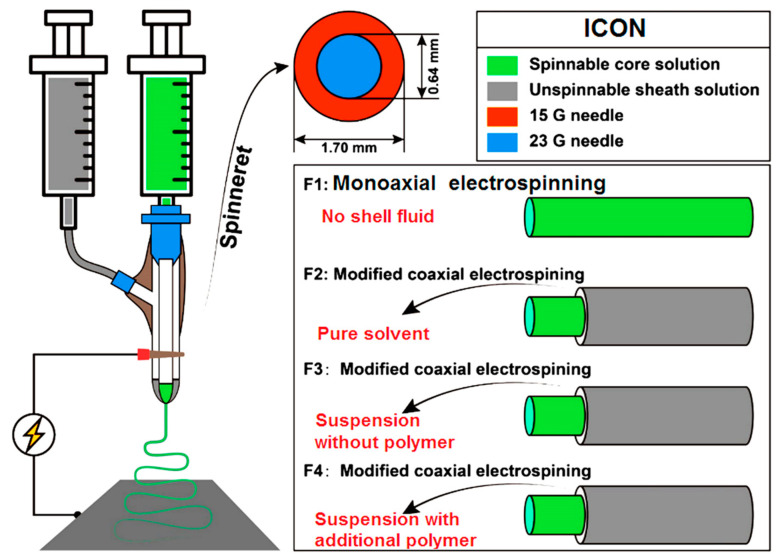
An illustration of the implementations of different kinds of modified coaxial electrospinning processes for creating medicated nanofiber-based orthopedic dressings.

Figure 2 is a diagram of the implementations of modified coaxial electrospinning using several unspinnable fluids, which include pure solvent as a shell fluid for creating nanofibers F2, suspensions containing only HAP nanoparticles and TA as the shell fluid for creating nanofibers F3, and suspensions containing HAP nanoparticles and TA, and a small content of PVP as the shell fluid for creating nanofibers F4. Meanwhile, a composite fenoprofen-PCL blended nanofibers F1 from single-fluid monaxial electrospinning was prepared for comparison.

**Figure 3:**
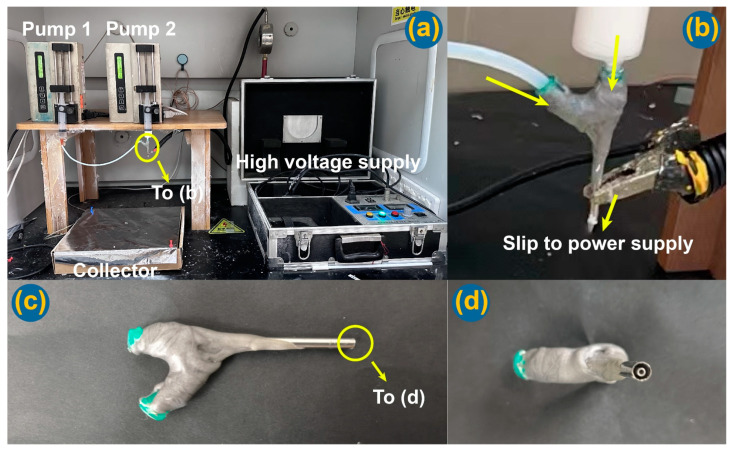
The implementations of electrospinning processes: (**a**) a whole digital image of the homemade electrospinning apparatus; (**b**) the connection of the concentric spinneret with the syringes holding the working fluids and high voltage supply; (**c**) an overall picture of the concentric spinneret; and (**d**) a high definition enlarged image of the spinneret’s outlet.

The implementations of electrospinning processes are presented in Figure 3. Figure 3a is a whole digital image of the homemade electrospinning apparatus, in which the spinneret was set up above the fiber collector. In the literature, many electrospinning apparatuses are arranged in a horizontal manner [17,83,84], which has few influences on the working processes and the resultant nanofibers. The connections of the spinneret with the two syringe pumps and the power supply are exhibited in Figure 3b. One syringe pump was directly connected with the spinneret to drive the shell working fluid, whereas the other pump pushing the core fluid was connected to the spinneret through Teflon tubing. The high voltage was transferred to the working fluid through an alligator clip. In an electrospinning apparatus, the spinneret is always the key element. In the investigation, the homemade spinneret was prepared by nesting a 23 G metal capillary (with an outer diameter of 0.64 mm) into a 15 G metal capillary (with an outer diameter of 1.7 mm) (Figure 2). The digital images of the homemade spinnerets are shown in Figure 3c,d. The spinneret was very light and convenient for setting up the whole working system. Additionally, the homemade coaxial spinneret had an arrangement in which the top of the core capillary was slightly projected out of the shell capillary. Its purpose is to effectively prevent the possible diffusion between the two working fluids and to provide a continuous and stable encapsulation of the core liquid by the shell fluid.

### 3.3. Morphology and Inner Structures of Nanofibers

**Figure 4:**
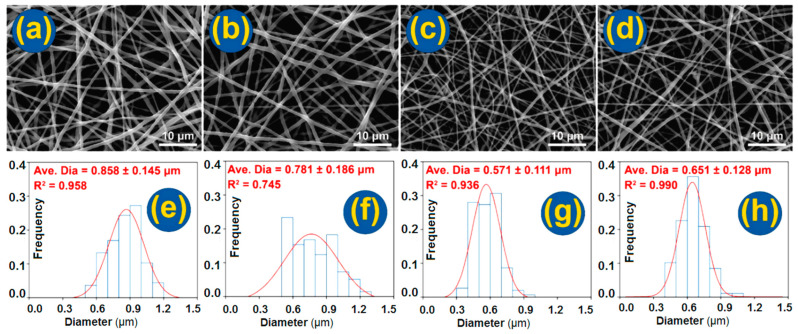
SEM images of the prepared nanofibers and their average diameters and size distributions: (**a**,**e**) F1; (**b**,**f**) F2; (**c**,**g**) F3; and (**d**,**h**) F4.

The morphologies of the four kinds of prepared nanofibers, i.e., the monoaxial fenoprofen-PCL composite nanofibers F1 from the single-fluid blending process, and the three nanofibers (F2, F3, and F4) from the modified coaxial electrospinning processes with different types of shell working fluids, are presented in Figure 4. The average diameters of all four kinds of fibers were smaller than 1.0 μm. All the nanofibers had a straight linear morphology without the negative beads-on-a-string or spindles-on-a-string morphologies. The shell fluid flow rate was much lower than the core fluid flow rate and thus did not degrade the whole modified coaxial electrospinning processes to the creation of beads or spindles and even to a coaxial electrospraying process. The influences of various amounts of shell-working fluids will be further investigated systematically in the future.

In comparisons between F1 and F2, the latter clearly had a smaller diameter and a narrower diameter distribution (781 ± 186 nm) than the former (858 ± 145 nm), although both were fenoprofen-PCL nanocomposites. The solvent as a shell working fluid could exert positive influences on the working processes and the quality of resultant nanofibers. On the one hand, the shell solvent was able to prevent the premature formation of semisolid substances on the surface of fluid jets. On the other hand, the shell solvent could act as a bridge for the evaporation of solvent in the core fluid to the atmosphere. Thus, the shell solvent could provide a longer period of drawing under the electrical field in a stabler and more robust manner, by which high-quality nanofibers with a smaller diameter and a narrower size distribution could be created. Similarly, the core–shell nanofibers F3 and F4 had a narrower diameter distribution than nanofibers F1, with an R^2^ value of 0.936 and 0.990, respectively. The addition of 5% PVP should result in a thicker shell of nanofibers F4 than that of nanofibers F3. The nanofibers F4 had an average diameter of 651 ± 128 nm, slightly larger than F3 (571 ± 111 nm).

**Figure 5:**
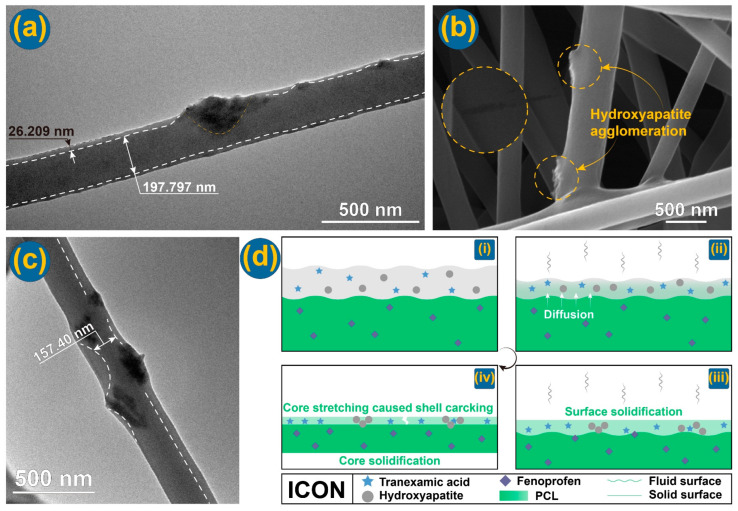
Inner structures and microformation mechanism: (**a**) TEM image of F3; (**b**) SEM image of F3—the dotted circles indicate a surface crack and two HAP aggregates, respectively; (**c**) TEM image of F4; and (**d**) an illustration of microformation mechanism of core–shell nanofibers F3, in which i, ii, iii and iv represent the progressive procedures.

The TEM image of F3 shows an obvious core–shell fibrous structure with nanoparticles clinging to the core section (Figure 5a). An enlarged SEM image in Figure 5b concurs the presence of HAP particles on the surface of core–shell nanofibers, as indicated by the two small yellow dotted circles. Interestingly, a crack is found on the enlarged surface image (indicated by a big yellow dotted circle in Figure 5b). These strange phenomena suggested that the microformation mechanism of the core–shell nanostructure by the modified coaxial electrospinning and with a suspension as a shell fluid was complicated. In another work, Zhou et al. [85] prepared a CA@ciprofloxacin (CIP) nanofiber through a modified coaxial electrospinning with CIP solution as a shell working fluid. Similarly, a strange phenomenon is that CIP nanocrystals were coated on the surface of CA fibers. Although there is no polymer in the shell fluid for preparing nanofibers F3, the solvent in the shell section might induce the diffusion of PCL molecules to form a thin shell layer, which had a thickness of 26.209 nm. The core section had an estimated diameter of 197.797 nm. As for the nanofibers F4, their TEM images are exhibited in Figure 5c. Similarly, with nanofibers F3, the core–shell nanostructures and the distributions of HAP particles on the surface of nanofibers can be observed.

Based on the observed cracks on the surface of nanofibers, the microformation mechanism can be suggested, which is diagrammed in Figure 5d. Initially, the shell suspension of TA and HAP nanoparticles was able to match the spreading of the core solution of PCL and fenoprofen due to the abundant solvents (Figure 5d(i)). Later, during the electrical drawing processes, both the shell suspension and the core solution evaporated their solvents into the atmosphere, as shown in Figure 5d(ii). However, the solvent molecules in the core solution must first pass through the shell section before they go into the surroundings. Meanwhile, the shell section would be solidified before the core section due to direct contact with the environment (Figure 5d(iii)). Thus, it is inevitable that some solvent molecules will be trapped in the core sections, which causes the core sections to have a delayed drying process. It is just the late drying of core sections that the previously solidified shell section will experience an inner additional drawing forces. This should be the reason for the formation of cracks on the solid fibers’ surfaces (Figure 5d(iv)).

### 3.4. Physical States and Compatibility

**Figure 6:**
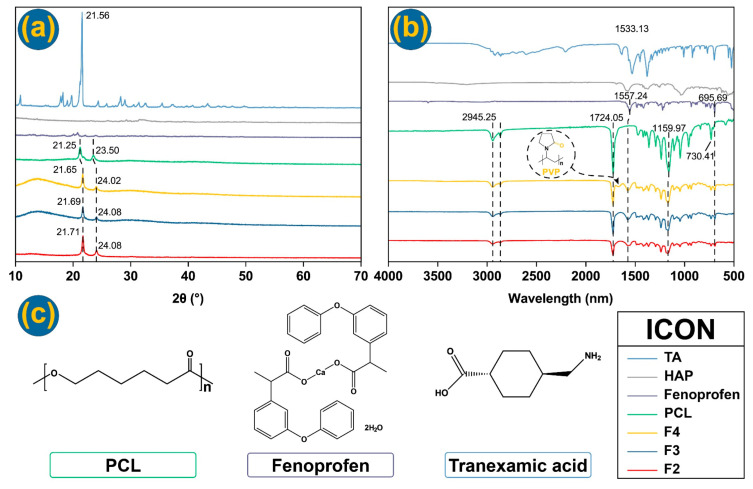
Physical state and compatibility: (**a**) XRD and (**b**) FTIR curves of raw materials and nanofibers; (**c**) structural formula of raw materials.

The physical states of raw materials and nanofibers can be determined using XRD, which is important for the presence and exerting functional performance of the loaded active ingredients [86,87,88,89]. From XRD patterns in Figure 6a, the untreated PCL has two typical sharp peaks at 21.25° and 23.50°. This can be attributed to the inherent crystallinity of the raw PCL powders. Notably, the nanofibers without TA (nanofibers F2) displayed similar peaks, indicating that these peaks were not contributed by TA. This suggests that TA is distributed in the core–shell nanofibers (nanofibers F3 and F4) in an amorphous state without any phase separations or formation of TA nanoparticles during storage. Similarly, HAP was also present in an amorphous state within the core–shell nanofibers F3 and F4. This is beneficial for promoting the osteogenic differentiation of adipose mesenchymal stem cells and facilitating bone regeneration, making it an ideal structure for precise and targeted bone tissue repair. Regarding fenoprofen, it was uniformly dissolved with PCL in the core fluid. The rapid drying process ensured that the homogeneous distribution in the working solutions was maintained in the solidified nanofibers. As a result, fenoprofen molecules were evenly dispersed throughout the PCL matrix at a molecular level without the formation of fenoprofen crystal nuclei or potential crystal growth.

The compatibility between the three small molecular drugs and the polymer fibers is important for both the stability of nanofibers and their functional performances [90,91,92], which can be disclosed through FTIR spectra (Figure 6b) and their molecular formula (Figure 6c). In FTIR tests, different groups can be observed. PCL demonstrates absorbance peaks at 1361.50 cm^−1^, 1419.35 cm^−1^, and 1471.90 cm^−1^ arising from bending vibrations of -CH_2_, at 2866.18 cm^−1^ and 2944.77 cm^−1^ (symmetric and asymmetric -CH_2_ stretching), and at 1236.63 cm^−1^ (-COO vibration). In the TA spectrum, some absorbance peaks, such as 1009.55 cm^−1^, are contributed by the C-N bond. Peaks at 1531.69, 1635.34, and 2209.54 cm^−1^ are contributed by C=O. The group C-H stretching vibration contributes 2863.77 cm^−1^ and 2920.18 cm^−1^. In the fenoprofen FTIR curve, the asymmetric and symmetric stretching of -O-C=O contributes to the peak absorbance at 1557.24 cm^−1^, which can be observed from the FTIR curves of three fibers [93]. The peak at 3597.56 cm^−1^ is from the hydrated compound, and this peak has been removed from fiber curves. It can be concluded that nanofibers demonstrate favorable drug-carrying properties, indicating good compatibility between the drugs and polymers used. This suggests that when these materials are utilized as orthopedic dressings, the three loaded drugs are expected to coexist stably, ensuring their effectiveness in a synergistic manner.

### 3.5. Wettability of Fibrous Membranes

**Figure 7:**
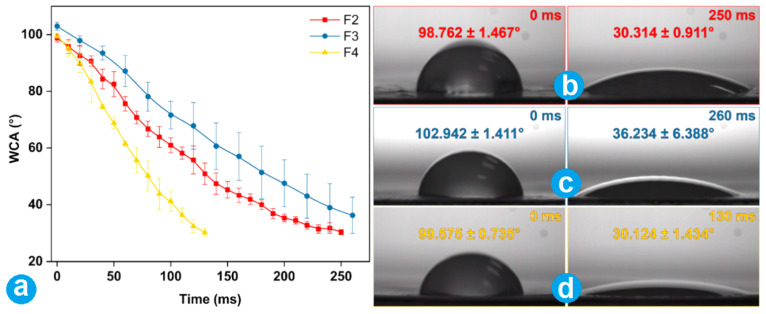
WCA results: (**a**) the change profiles of three kinds of nanofibers with time; (**b**–**d**) the initial and final images of water droplets on the nanofibers F2, F3, and F4, respectively.

Three prepared nanofibers (F2~F4) performed fine wettability. The WCA tests showed that they all had an angle around 100° initially. But, all WCAs were reduced to lower than 40° quickly in 300 ms (Figure 7a). PCL is a hydrophobic material with good biocompatibility, is biodegradable, and can provide a desired drug sustained release profile due to its insolubility. Furthermore, the core-loaded drug fenoprofen has a fine hydrophilicity. Thus, the addition of fenoprofen not only improved the nanofibers’ hydrophilicity but also ensured a diffusion-controlled release behavior. Figure 7b–d show that the nanofiber membranes F2, F3, and F4 dropped from their initial angle (larger 100°) to an angle smaller than 40°, respectively. Nanofibers F4 (Figure 7d) took the shortest time of 130 ms, which should be attributed to the presence of PVP K30 in their shell sections. PVP is a hydrophilic polymer and is applied in many composite materials to increase their hydrophilicity [11]. As a result, a PVP K30-loaded shell of nanofibers F4 granted them the best hydrophilic property in three fibrous membranes. For clinic orthopedic dressing applications, the fine hydrophilic property is a favorite element for achieving a better hemostatic application.

### 3.6. In Vitro Release Profile of Fenoprofen

**Figure 8:**
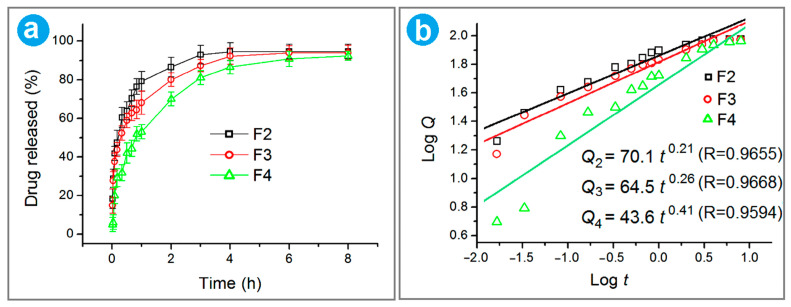
In vitro release profile of fenoprofen: (**a**) in vitro release curve of fenoprofen from F2, F3, and F4; (**b**) the fitting curves of the three kinds of nanofibers according to Ritger–Peppas model.

Over the past few decades, numerous studies have been conducted on the sustained release of water-soluble drugs. In order to achieve this purpose, almost all materials processing methods have been introduced into this application. During an electrospinning process, polymers, both with water-resistant and biodegradable properties, are often chosen to encapsulate drug molecules. Drug molecules can be released through diffusion, erosion, or a combination of them, which is initially determined by the properties of host polymeric matrices. Here, PCL has many merits, like high mechanical properties, biocompatibility, and fine filament-forming capability to support its potential medical applications. In this study, the polymer PCL was selected as the polymer matrix had a “two birds with one stone” effect, i.e., filament-forming for a successful coaxial preparation and a host hydrophobic polymer for a sustained release of fenoprofen. To further understand the effect of the nanofiber membrane on the action of fenoprofen, the release rates of fenoprofen from nanofibers were studied. A standard curve of fenoprofen at 271 nm is *A* = 0.00356 + 0.00617 *C* (R^2^ = 0.99988), where *A* and *C* represent absorbance and drug concentration (mg/mL), respectively. According to the achieved fenoprofen release profile shown in Figure 8a, 76.79%, 68.17%, and 53.06% of the loaded drug were released in the first hour from nanofibers F2, F3, and F4, respectively. F2 showed the most severe initial burst release, which should be attributed to the drug molecules distributed on the surface of nanofibers and their fine solubility. Although F4 had soluble PVP K30 on the surface, the initial burst release was remarkably reduced. This should be the result of two aspects. One is that the surface of F4 had no drug molecules. The other is that the core fenoprofen-PCL composites should have a more compact surface to retard the dissolution and release of fenoprofen from the inner section. During the modified coaxial electrospinning, the shell PVP K30 working fluid was able to play an important role in promoting the gradual evaporation of the inner core solvent and, in turn, the dense accumulation of PCL molecules on the core sections’ surface.

Later, the drug release rate began to flatten out, and the drug encapsulated in the fiber was controlled to be released in a relatively stable and continuous manner. After 6 h, all three kinds of nanofibers were able to release over 90% of the loaded fenoprofen. Drug dissolution kinetics is an important method for analyzing and understanding the drug release mechanism. In order to further study the effect of modified coaxial nanofibrous structures on drug molecule release behavior, the Ritger–Peppas equation was used to disclose the mechanism of drug molecule release in the dissolved medium. The curves fitted by the Ritger–Peppas model [94] are included in Figure 8b. The equations for nanofibers F2, F3, and F4 are Q_2_ = 70.1 *t*^0.21^ (R = 0.9655), Q_3_ = 64.5 *t*^0.26^ (R = 0.9668), and Q_4_ = 43.6 *t*^0.41^ (R = 0.9594), respectively. The n values of 0.21, 0.26, and 0.41 are all smaller than 0.45, indicating that the release mechanism of fenoprofen from all the nanofibers was a typical Fickian diffusion one. This can be anticipated from the insoluble property of host polymeric matrix PCL and the soluble property of fenoprofen. For nanofibers F3 and F4, controlled hemostasis is achieved through the rapid release of TA from the nanofiber surface. Simultaneously, the rapid release of HAP promotes the repair of bone hyperplasia. Following these immediate actions, the sustained and gradual release of fenoprofen from the nanofibers becomes beneficial in relieving inflammation and alleviating pain in the injured bone tissue. This sequential release mechanism ensures a comprehensive and effective treatment approach for bone injuries.

### 3.7. In Vitro Hemostatic Time Test

**Figure 9:**
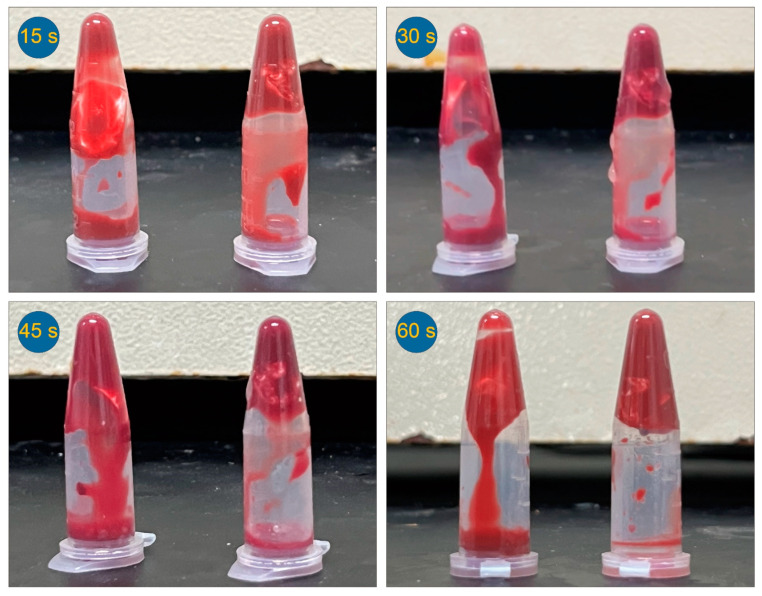
Hemostatic effect of different membranes at predetermined times: F2 (left in each picture) and F3 (right in each picture).

To avoid hypotension, anemia, or other complications caused by hemorrhage during orthopedic surgery, TA has been broadly explored to promote hemostasis in orthopedic trauma surgery. In order to evaluate the hemostatic effect of the fiber membranes, the whole blood coagulation time of nanofibers loaded with TA and without HA (F3 and F2) was tested. By comparing the clotting time of hemostatic in vitro of different fibrous membranes, the hemostatic properties of fibrous materials were disclosed in Figure 9. Theoretically, the presence of TA is more likely to promote blood clotting for the same amount of time. The comparison of the coagulation conditions of nanofiber membranes F2 and F3 within 60 s can reflect this fact elaborated above more intuitively. A comparison between F2 and F3 shows that F3 comprising TA has a good performance in clotting anticoagulant blood, and they would be fully clotted after 60 s. However, the same process by using F2 should be quite slow. Anticoagulant blood remained in a flow state after 60 s. This is because the hemostatic drug TA is present in the nanofiber membrane F3. After the occurrence of coagulation, the fiber membrane F3 was aggregated to form a larger size, which may be caused by the adhesion of some platelets to the nanofiber membrane under the action of TA. It also further proves the hemostatic effect of TA in F3. This difference between F2 and F3 displays that TA has a fast-release profile and performs its role in hemostasis.

Electrospun nanofibers are widely recognized as excellent candidates for hemostatic applications due to their distinctive properties, including high porosity, small diameter, and large surface area [95,96,97]. This investigation has revealed that the incorporation of drugs and the strategic manipulation of their distribution within the nanofibers can significantly enhance their hemostatic capabilities. Furthermore, the simultaneous loading of multiple active ingredients imparts the nanofibers with diverse functional performances, further expanding their potential applications in wound management. Electrospinning is a flexible top–down technique with a broad spectrum of regulatable parameters [98,99,100,101,102]. Based on the concept disclosed here, many new possibilities for developing novel hemostatic nanomaterials and other fantastic applications of electrospun architectures [103,104,105,106,107] can be anticipated.

## 4. Conclusions

In this investigation, with a pure solvent, a suspension without polymer, and a suspension with PVP as a shell working fluid, three modified coaxial electrospinning and single-fluid electrospinning of the core fluid were successfully conducted, and four electrospun fibers were prepared. SEM and TEM evaluations indicated that the prepared fibers all had a linear morphology with a diameter smaller than 1.0 μm. HAP could be well dispersed on the surface of nanofibers, whereas TA and fenoprofen were uniformly distributed in the shell and core chambers of the electrospun core–shell nanofibers, respectively. HAP exhibited minimal agglomeration and adhered clearly to the core sections of the nanofibers. XRD indicated that fenoprofen and TA existed in the electrospun nanofibers in an amorphous form, and FTIR results suggested good compatibility between the host polymer and the incorporated drugs. Wettability tests showed that all kinds of prepared nanofibers displayed favorable hydrophilic properties. In vitro dissolution tests revealed a sustained release of fenoprofen over 6 h via a typical Fickian diffusion mechanism. Hemostatic tests indirectly indicated the rapid release of TA from the fibrous membrane within 60 s. Based on the above results, it can be suggested that the electrospun fenoprofen/PCL@TA/HAP/PVP nanofibers F4 exhibit the most favorable characteristics, including rapid release of TA and HAP, as well as extended release of fenoprofen, enabling a synergistic and sequential therapeutic effect. Consequently, these nanofibers show great potential as orthopedic hemostatic dressings.

## Figures and Tables

**Table 1:** Parameters for the preparations of electrospun nanofibers.

NO.	Electrospinning	Core (*w*/*v*)	Shell (*w*/*v*)	Flow Rate (mL/h)	Morphologies
				Core/Shell	
F1	Uniaxial	Solution A	--	1.2/0	Monoaxial fiber
F2	Modified coaxial	Solution A	TFE	1.2/0.3	Monoaxial fiber
F3	Modified coaxial	Solution A	Solution B	1.2/0.3	Core–shell nanofiber
F4	Modified coaxial	Solution A	Solution B + 5% (*w*/*v*) PVP K30	1.2/0.3	Core–shell nanofiber

Solution A: 10% (*w*/*v*) PCL and 4% (*w*/*v*) fenoprofen in DCM, EtOH (9:1, *v*/*v*). Suspension B: 1% TA and 1% HAP in TFE (*w*/*v*).

## Data Availability

The data supporting the findings of this manuscript are available from the corresponding authors upon reasonable request.
